# Starvation Ketoacidosis in a Young Healthy Female After Prolonged Religious Fasting

**DOI:** 10.7759/cureus.39962

**Published:** 2023-06-05

**Authors:** Ramanathan Ramesh, Arulmoly Kanagasingam, Sithy Sabrina, Uthayakumar Anushanth

**Affiliations:** 1 Medicine, Teaching Hospital-Batticaloa, Batticaloa, LKA

**Keywords:** hypoglycemia, euglycemia, metabolic acidosis, ketoacidosis, starvation

## Abstract

Ketone bodies are important energy sources for the body and are produced by the liver when the body is in a deficiency state of glucose, which is used in the peripheral tissues to provide energy. There are several ketone bodies that are produced by the liver, of which two are important: acetoacetate and beta-hydroxybutyrate. Even though ketone bodies are always present in the body, they are minimal when a person is not fasting. Ketone bodies are produced by the oxidation of fatty acids to fulfill the metabolic needs of tissues, especially the brain. The biochemical reactions of forming ketone bodies are triggered by a lack of insulin and an elevated glucagon level in the blood. Both cause unopposed lipolysis and free fatty acid oxidation resulting in the production of ketone bodies and eventually high anion gap metabolic acidosis. We present a case of young healthy female who presented with euglycemic ketoacidosis after involving prolonged fasting for her religious ceremony. She also physically exerted quite more during her fasting. With a detailed history and excluding other possibilities, we made the diagnosis of starvation ketoacidosis. She improved well with the treatment and established her pre-morbid condition in our review.

## Introduction

There are three important causes of ketoacidosis, such as diabetic ketoacidosis, alcoholic ketoacidosis, and starvation ketoacidosis [1-3]. All the above causes can be suspected or diagnosed initially with history. Carbohydrate depletion is the initial driving force for ketogenesis in starvation ketoacidosis. Mild ketosis (1 mmol/l) generally develops after 12 to 14 hours of fasting, but if the starvation continues, the level of ketone rises to 8 to 10 mmol/l [4,5].

Euglycemic diabetic ketoacidosis usually presents with severe metabolic acidosis, while starvation ketoacidosis typically presents with a pH above 7.3 [6]. Clinicians should remember to form a list of differential diagnoses when evaluating a patient with acute metabolic acidosis.

Unlike diabetic ketoacidosis, patients with fasting ketosis release insulin when carbohydrate is replaced exogenously. They are also producing elevated levels of glucagon and reducing glycogen stores. These hormones lead to lipolysis, which causes the formation of ketones for fuel. The administration of insulin in this state causes hypoglycemia. Once treated with adequate carbohydrates, the insulin level will increase and counter-regulatory hormone levels will be reduced, and ketosis will be resolved.

Here we are presenting a case of a 32-year-old previously healthy lady who presented with starvation ketoacidosis following prolonged religious fasting. This is a unique case of starvation ketoacidosis in relatively young healthy patients.

## Case presentation

A 32-year-old woman, a previously healthy mother, presented with several episodes of vomiting and a reduced level of responsiveness. She was relatively healthy until she was admitted with the above symptoms following prolonged religious fasting with very minimal calorie intake. She was apparently taking one meal per day with a small amount of water for sixteen days before admission. Even though she had not been eaten anything for the last 36 hours, she had taken only two to three sips of water during that period prior to admission. Her relatives denied a history of fever, headache, photophobia, phonophobia, altered behavior, or abnormal limb movements suggestive of seizures. There was no history of limb weakness or abnormal gait. Her past medical history was not significant. There was no past or family history of epilepsy or psychotic illness noted. On the day of hospital admission, following several episodes of vomiting, she became restless and agitated, and her consciousness was reduced.

On admission at the emergency department, her Glasgow Coma Scale (GCS) was 10/15 (E-3, V-2, M-5). Her oxygen saturation was 94% in room air, her heart rate was 126 beats per minute, and with a thready pulse, her blood pressure was 100/60 mmHg. Her body mass index (BMI) was 24.3 kg/m^2^. She was drowsy, dehydrated, and afebrile, and her peripheries were cold. There were no signs of neck stiffness, skin rashes, lymphadenopathy, peripheral stigmata of chronic liver cell disease, or chronic kidney disease observed. Her respiratory and abdominal examinations were unremarkable. Limited examination of neurology, including pupillary sizes and reactions, was normal other than reduced consciousness. Her ECG showed sinus tachycardia only, without any other abnormalities. Her brain computed tomography and chest imaging were normal. Her other investigation results are as follows (Table 1).

**Table 1:** Investigation results during the ward stay. FBC: full blood count, WBC: white blood cells, Hb: hemoglobin, PLT: platelet, LFT: liver function test, AST: aspartate aminotransferase, ALT: alanine aminotransferases, ALP: alkaline phosphatase, ESR: erythrocyte sedimentation rate, CRP: C reactive protein, RFT: renal function test, BU: blood urea, SE: serum electrolytes, UFR: urine full report, RBC: red blood cells, RBS: random blood sugar.

Investigations	Day 1	Day 2	Day 4	On discharge
FBC-WBC/mm^3^	25.1 × 10^3^	9.8 × 10^3^	8.4 × 10^3^	7.8 × 10^3^
Neutrophil (%)	78	68	65	62
Lymphocytes (%)	18	29	28	18
Eosinophils (%)	2.1	3.5	2.4	7.8
Hb (g/dl)	14.4	12.4	11.2	11.1
PLT (mm^3^)	450	320	264	188
ESR	22		18	
CRP (mg/l)	28.4	22	17	16
LFT-AST (U/L)	42			44
ALT (U/L)	23			26
ALP (U/L)	46			54
Albumin (g/l)	34			32
Bilirubin-total (µmol/l)	13			15.2
Direct bilirubin (µmol/l)	7.8			8.2
Indirect bilirubin	5.2			7.0
RFT-BU (mmol/l)	6.9	5.2	3.8	1.9
Serum creatine (µmol/l)	190	134	88	68
Serum Na^+^ (mmol/l)/serum K^+^ (mmol/l)	144 5.6	133 3.2	142 3.3	138 3.8
UFR-pus cells	1-2	5-6		
RBC	Nil	1-4		
Protein	Trace	Nil		
Organism	Nil	Nil		
Urine ketone body	Positive		Negative	Negative
Serum ketone-total (µmol/l)	1062.4			
Acetoacetate	379.1			
Beta-hydroxybutyric acid	683.3			
RBS (mg/dl)	92	114	108	132

As the patient's HCO_3_ was very low at admission, we did serial ABG when she had been on treatment (Table 2). The patient was initially resuscitated at the emergency treatment unit (ETU) with good hydration and an intravenous dextrose infusion. 

**Table 2:** Serial ABG analysis on admission and throughout the ward stay. PCO_2_: partial pressure of carbon dioxide, PO_2_: partial pressure of oxygen, HCO_3_: bicarbonate.

Arterial blood gas analysis (ABG)	Day 1	Day 2	Day 4	On discharge
PH	7.324	7.34	7.404	7.45
PCO_2_ (mmHg)	9.9	15.7	16.8	22
PO_2_ (mmHg)	49.5	55	55	85
HCO_3_ (mmol/L)	5.2	8.6	10.6	19
Lactate (mmol/L)	10.9	10.2	8.5	2.4
Anion gap	32	28	16	

## Discussion

She was 32 years old, previously healthy, and presented with one day history of illness. She had suffered from several episodes of vomiting and became agitated, followed by reduced responsiveness. When we analyzed the detailed history from family members and later with the patient, the only significant history was that she had been in continuous religious fasting for the last 16 days, which she intensified for the last 36 hours before being presented to the hospital. There were no significant comorbidities or hospital admissions in the past. Her initial evaluation made us make a list of differential diagnoses, including meningoencephalitis, Wernicke’s encephalopathy followed by severe vomiting, and diabetic ketoacidosis as a first presentation of diabetes mellitus.

On initial evaluation in the emergency department, her capillary blood sugar and later her random venous blood sugar turned out to be normal. In the context of her history, we made a tentative diagnosis of euglycemic ketoacidosis due to prolonged fasting. However, we had some other possible differential diagnoses as well. Because her presentation was very acute onset, we suspected poisoning like yellow oleander or cerebra manga or snake bite, as they are very common in this part of the country. Even though she was young and there were no risk factors, we considered the stroke also as a possible differential diagnosis. We continued our diagnostic work-up to arrive at the diagnosis. As her capillary blood sugar was 92 mg/dl, we suspected euglycemic ketoacidosis due to prolonged fasting rather than diabetes ketoacidosis.

Blood samples were taken for serum ketone body level, ABG, and all other relevant investigations. Her ABG showed metabolic acidosis with a low HCO_3_ level of 5.2 mmol/l, and her urine ketone body was also positive. Importantly, her serum ketone body quantitative assessment turned out to be positive, and we almost made the diagnosis at that point. We initiated the resuscitation with continuous intravenous dextrose infusion and correction of hypovolemia with normal saline. Intravenous infusion of HCO_3_ was also given at the emergency department to correct the acidosis. As it was difficult to exclude Wernicke’s encephalopathy, we started IV thiamin 100 mg daily as well. Blood and urine cultures have been taken, and she has been started on intravenous ceftriaxone and acyclovir as an initial broad-spectrum antibiotic and antiviral therapy, respectively. Even though her brain imaging (non-contrast computed tomography) was normal, we could not proceed with the lumbar puncture as her clinical condition did not permit.

After initial management, she was transferred to the ward for further continuous care. She significantly improved in the ward from her acute illness. The next day, she completely recovered, both hemodynamically and neurologically. However, we continued our management with dextrose infusion while monitoring her capillary blood sugar and ABG until her oral diet came back to normal. Since the patient was completely recovered and all her blood investigations, including her electrolytes, calcium, magnesium, and renal functions, became normal, we excluded the evidence of sepsis and meningoencephalitis and did not proceed with the lumbar puncture test.

Even though starvation keto acidosis is not a common condition, early suspicion from the clinical evaluation and subsequent initial treatment is crucial. If it is not managed properly, it can be fatal even. It’s important to check for starvation ketoacidosis in a patient with unexplained metabolic acidosis and euglycemia or hypoglycemia, particularly in pregnant mothers, neonates, the malnourished, the underweight, people with eating disorders, and people who follow the ketogenic diet, as they are more prone to get it [7].

In our patient, her BMI was 24.3 kg/m^2^, and there was no history of any eating disorders or psychotic illness, but with strict fasting, she had physically exerted herself a lot at temple ceremonial events. So, it is important to suspect in healthy people, as in this case, we had an experience.

The management of starvation ketoacidosis is mainly to replace glucose, as hypoglycemia is the trigger factor for the formation of the ketone body in starvation ketoacidosis. Management consists of a combination of intravenous dextrose and appropriate replacement of electrolytes with cautious monitoring [8-10]. The early diagnosis and prompt treatment made our patient's quick recovery. She got treatment at the ward for five days and was discharged after the complete recovery of clinical and biochemical means. We reassessed the patient after one week of discharge, and she was back to her normal life. Her illness was well explained throughout the hospital stay to the patient and the next of kin, including preventive measures in the future.

## Conclusions

This case report is a good example to remind a clinician to screen for starvation ketoacidosis when a patient presented with unexplained metabolic acidosis. It’s important to check the blood sugar and blood ketone body level in the initial resuscitation. Starvation ketoacidosis can be easily missed when a patient presented with normal or low blood sugar and ketoacidosis. Even though starvation ketoacidosis is uncommon in healthy adults, as in our case, it can happen, and a thorough history and risk identification of developing starvation ketoacidosis will be helpful in making the diagnosis.
